# Ozonometer

**Published:** 1859-02

**Authors:** 


*Ozonometer.*—Dr. Lankester exhibited to the Chemical Section of the British Association for the advancement of Science, at its late meeting in Leeds, an instrument for measuring the constant intensity of ozone. This instrument consisted of two small rollers included in a box, which were moved by means of ordinary clockwork. Over the roller a strip of paper, prepared with iodide of potassium and starch is allowed to revolve, the paper becoming exposed to the air for an inch of its surface in the lid of the box Twenty-four inches of paper pass over the rollers in the course of twenty-four hours, and thus registers, by its color, the intensity of the action of ozone in the atmosphere. By this instrument, the intensity of the ozone for every hour in the twenty-four could be registered, and *minima* and *maxima*, with an average, ascertained. The register of ozone could also be compared with those of the anemometer, and the relation of ozone to the direction and force of the wind ascertained. Dr. Lankaster pointed out the importance of ascertaining the presence of ozone, on account of its undoubted relation to health. He drew attention to a series of tables which had been drawn up from the registrations of anemometer made at London, Blackheath, and Felixstow, on the coast of Suffolk. From these it was seen that the relation of these three places were 0, 22, and 55. The instrument acted also as a clock, and the time could be accurately marked upon the ozonized paper.

Mr. Marshall made some remarks on his own observations during the last twelve months, and stated that he had not been able to discover, though assisted in the investigation by medical gentlemen, that there was any obvious connection between ozone and the state of health.—*British Med. Journ.*, Oct. 16, 1858, from *The American Journ. Med. Sciences.*

